# The Effect of Electroporation of a Lyotroic Liquid Crystal Genistein-Based Formulation in the Recovery of Murine Melanoma Lesions

**DOI:** 10.3390/ijms160715425

**Published:** 2015-07-08

**Authors:** Corina Danciu, Szilvia Berkó, Gábor Varju, Boglárka Balázs, Lajos Kemény, István Balázs Németh, Andreea Cioca, Alexandra Petruș, Cristina Dehelean, Citu Ioan Cosmin, Elena Amaricai, Claudia Crina Toma

**Affiliations:** 1 Department of Pharmacognosy, “Victor Babes” University of Medicine and Pharmacy, 300041 Timisoara, Romania; 2 Department of Pharmaceutical Technology, University of Szeged, Szeged 6720, Hungary; E-Mails: berko@pharm.u-szeged.hu (S.B.); balazs.boglarka@pharm.u-szeged.hu (B.B.); 3 Dr. Derm Clinic of Anti-Aging Dermatology, Aesthetic Laser and Plastic Surgery, Budapest 1026, Hungary; E-Mail: g.varju@drderm.net; 4 Department of Dermatology and Allergology, University of Szeged, Szeged 6720, Hungary; E-Mails: kl@mail.derma.szote.u-szeged.hu (L.K.); nemeth.istvan.balazs@med.u-szeged.hu (I.B.N.); 5 Department of Pathology, “Iuliu Hatieganu” University of Medicine and Pharmacy, 400006 Cluj-Napoca, Romania; E-Mail: cioca_andre@yahoo.com; 6 Department of Anatomy, Physiology and Pathophysiology, “Victor Babes” University of Medicine and Pharmacy, 300041 Timisoara, Romania; E-Mail: alexandra.petrus@umft.ro; 7 Department of Toxicology, “Victor Babes” University of Medicine and Pharmacy, 300041 Timisoara, Romania; E-Mail: cadehelean@umft.ro; 8 Department of Obstetrics and Gynecology, “Victor Babes” University of Medicine and Pharmacy, 300041 Timisoara, Romania; E-Mail: citu.ioan@umft.ro; 9 Department of Rehabilitation, Physical Medicine and Rheumatology, “Victor Babes” University of Medicine and Pharmacy, 300041 Timisoara, Romania; 10 Department of Pharmacognosy, Western University “Vasile Goldis”, 310025 Arad, Romania; E-Mail: claudiatoma2004@yahoo.com

**Keywords:** genistein, lyotropic liquid crystals, electroporation, murine melanoma

## Abstract

A lamellar lyotropic liquid crystal genistein-based formulation (LLC-Gen) was prepared in order to increase the aqueous solubility of the lipophilic phytocompound genistein. The formulation was applied locally, in a murine model of melanoma, with or without electroporation. The results demonstrated that, when the formulation was applied by electroporation, the tumors appeared later. During the 21 days of the experiment, the LLC-Gen formulation decreased the tumor volume, the amount of melanin and the degree of erythema, but when electroporation was applied, all these parameters indicated a better prognosis even (lower tumor volume, amount of melanin and degree of erythema). Although hematoxylin–eosin (HE) staining confirmed the above events, application of the LLC-Gen formulation by electroporation did not lead to a significant effect in terms of the serum concentrations of the protein S100B and serum neuron specific enolase (NSE), or the tissue expression of the platelet-derived growth factor receptor β (PDGFRβ) antibody.

## 1. Introduction

The most recent statistics regarding new melanoma cases in Europe show that an average of 47.2 males and 53.1 females per 100,000 investigated subjects were diagnosed with melanoma in 2012 [1]. The incidence varies within the countries of the European Union, with very high rates registered in Switzerland, Denmark, and Norway as compared with Greece, Bulgaria, Cyprus and Romania [2,3]. Melanin pigmentation in mammalian skin is under complex regulatory control by several pathways triggered by receptor-dependent and -independent mechanisms, hormonal, auto-, para-, or intracrine manner [4]. As the incidence of melanoma is increasing worldwide, researchers are investing considerable amounts of time and money in the search for an effective treatment for this dangerous disease [5,6,7]. However there is no reliable therapy for metastatic melanoma despite the progress made in the field [8].

Natural compounds, and especially plant secondary metabolites, have been studied for centuries for their complex therapeutic benefits [9]. Different types of cancer have revealed positive responses to certain natural compounds both *in vitro* and *in vivo*. Genistein (Gen) is a naturally occurring compound in the class of isoflavonoids. It is well known for its estrogeno-mimetic properties [10]. It has been intensively studied in cases of breast and prostate cancer [11,12]. Its efficacy has been shown in malignant melanoma. In an earlier study we observed that Gen decreases the tumor size, the metastasis potential and the level of melanization in a B16 mouse model of melanoma. However, the recovery of the skin lesions was impaired [13]. Those data led us to propose another approach to promote the recovery of murine melanoma lesions: electroporation of a lyotropic liquid crystal Gen-based formulation (LLC-Gen).

Lyotropic liquid crystal systems (LLC) have recently received increased attention in both the cosmetic and pharmaceutical fields [14]. They flow like liquids (hence the name liquid crystals) and in part maintain the ordered structure of crystalline solids [15]. The advantageous qualities of such formulations include their amphiphilic nature, the similarity to colloidal systems existing in living organisms, the various structures of liquid crystal states, and their thermodynamic stability [16]. Gen is a lipophilic compound that is practically insoluble in water, which can be a real problem in terms of formulation and bioavailability. LLCs display good solubilizing effects, sustained release and enhanced bioavailability of other lipophilic drugs [17].

We decided to attempt a new approach by applying this new LLC-Gen formulation locally with or without electroporation, a process characterized by structural changes in the cell membrane barrier and resulting reversible increase in transmembrane transport; practically, aqueous pores are created in lipid bilayers. These transitory changes, possible due to the application of high-voltage pulses, can be used to load different molecules into the cells [18,19]. Primarily designed for gene transfer, electroporation is nowadays used for a wide range of structures, such as antibodies, oligonucleotides, DNA, ions, drugs, *etc.* [20].

Thus, the aims of this study were to investigate whether lamellar LLC systems form a good base for Gen incorporation, and to analyze the effects of electroporation with such a formulation in a murine model of melanoma.

## 2. Results and Discussion

### 2.1. Polarization Microscopic Examinations

In the development of the dermal delivery, we prepared a LLC formulation that is able to suspend Gen at a concentration of 3%.

**Figure 1:**
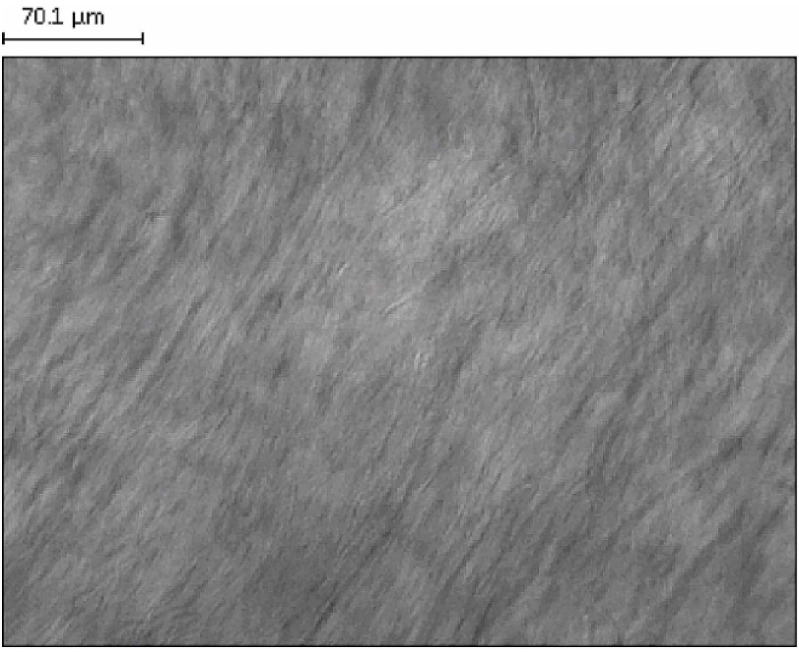
Polarizing microscopic examination of blank lyotropic liquid crystal systems (LLC) at a magnification of 20×.

Figure 1 presents a polarized microscopic picture of the developed LLC structure, revealing a lamellar LLC pattern with a characteristic ribbon structure in polarized light.

### 2.2. Rheological Investigations

**Figure 2:**
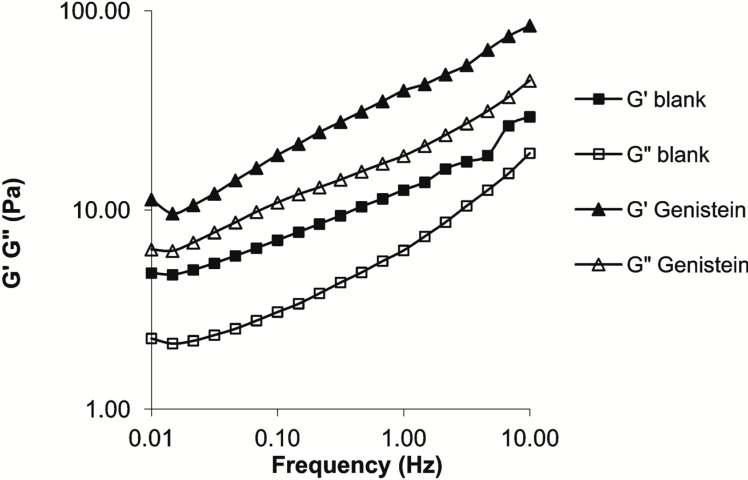
Rheological characterization of the blank and Genistein (Gen)-containing LLC formulations.

The characteristics of the LLC system include the frequency-dependent storage and loss moduli. In the investigated frequency range, the blank LLC system is more elastic than viscous. The solubilization of Gen in the LLC system led to a consistency increase (Figure 2).

**Figure 3:**
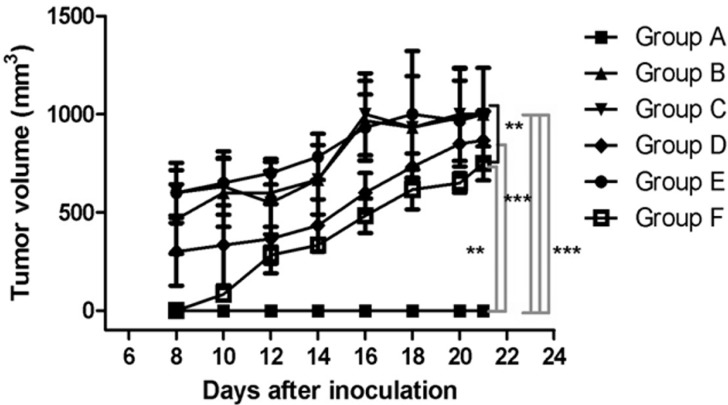
Tumor volumes (mm^3^) in the different experimental groups on day 21 of the experiment. ** *p* < 0.01, *** *p* < 0.001.

Melanoma was induced and the formulation was applied as indicated in the Experimental Section. In each of the inoculated mice, the volume of the tumor was observed to be increased, to an extent directly proportional to the number of days of the examination. Tumors appeared on day eight post-inoculation in both the treated and the untreated groups, with the exception of the mice in group F; in these mice, which were inoculated with B164A5 cells and treated with LLCs containing 3% Gen and electroporated for 6 min at high-voltage, the tumors appeared on day 10 post-inoculation. The mean tumor volume in group F was 83.33 ± 28.86 mm^3^, in contrast with 466.66 ± 208.16 mm^3^ in group B, 589.78 ± 204.67 mm^3^ in group C, 309.00 ± 207.81 mm^3^ in group D and 603.23 ± 264.57 mm^3^ in group E. Comparison of the curves corresponding to the different treatment approaches reveals that the LLC-Gen formulation decreased the tumor volume, but following electroporation of this formulation, the results were even better. On day 21 of the experiment, the tumor volumes were 1001.58 ± 409.26 mm^3^ in group B, 1000.86 ± 404.96 mm^3^ in group C, 866.66 ± 256.58 mm^3^ in group D, 999.87 ± 408.95 mm^3^ in group E and 751.00 ± 151.03 mm^3^ in group F. Significant results (*p ˂* 0.05) between the different experimental groups were found as shown in Figure 3.

**Figure 4:**
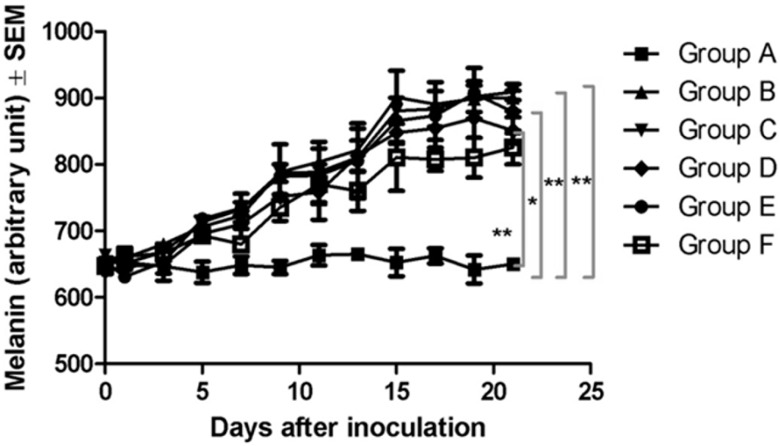
Melanin amounts (in arbitrary units (A.U.) as determined by the manufactured device) in the different experimental groups on day 21 of the experiment. * *p* < 0.05, ** *p* < 0.01.

During the 21 days of the experiment, noninvasive measurements of relative melanin pigmentation and the degree of erythema were performed every two days with the Courage-Khazaka Mexameter^®^ MX 18 Multiprobe Adapter System (MPA5). Curves corresponding to relative melanin pigmentation were plotted and differences relative to the blank group A were recorded starting from day five post-inoculation. The normal amount of melanin in the skin of the C57BL6J mouse varies in the interval 635–670 arbitrary units (A.U.). On day five post-inoculation, the interval increased to 695–720 A.U. Differences between the experimental groups in the amount of relative melanin pigmentation were observed on day 9: 645 ± 14 A.U. in group A, 789 ± 60 A.U. in group B, 788 ± 19 A.U. in group C, 752 ± 5 A.U. in group D, 782 ± 12 A.U. in group E and 735 ± 28 A.U. in group F. The curves presented in Figure 4 show that application of the LLC-Gen formulation to the skin resulted in a slight decrease in the amount of melanin, but when the formulation was applied by electroporation the level of pathological melanin was reduced significantly. On day 21 of the experiment, the results were 650 ± 13 A.U. in group A, 901 ± 21 A.U. in group B, 909 ± 17 A.U. in group C, 851 ± 28 A.U. in group D, 879 ± 45 A.U. in group E and 826 ± 36 A.U. in group F. Significant differences (*p ˂* 0.05) between the different experimental groups were found as shown in Figure 4.

**Figure 5:**
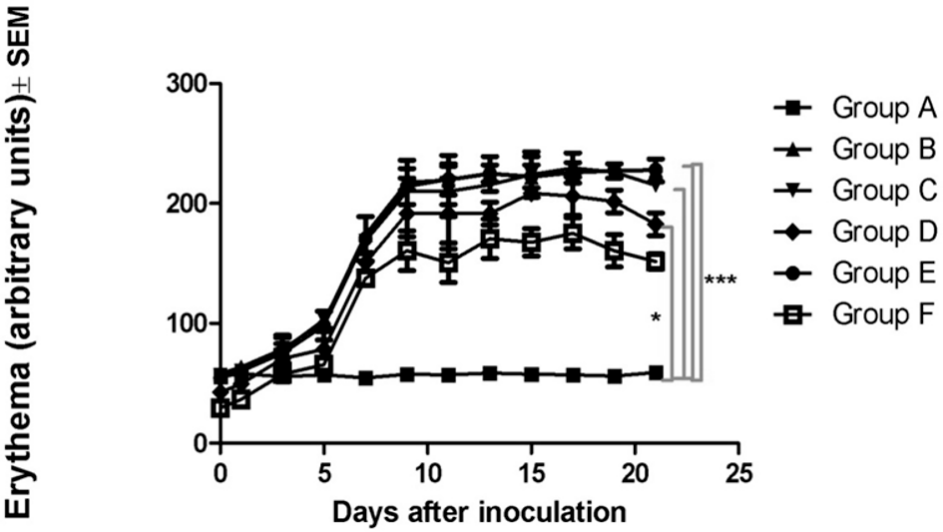
Erythema (in A.U. as determined by the manufactured device) in the different experimental groups on day 21 of the experiment. * *p* < 0.05, *** *p* < 0.001.

The MX18 device was also employed to determine the degree of erythema after the appearance of the tumors and to observe any changes that occurred after treatment. The normal values for the skin of the C57BL6J mouse are in the range 50–60 A.U. Increased levels, corresponding to erythema, were observed from day nine post-inoculation. The recorded levels on day nine were 58 ± 2 A.U. in group A, 218 ± 26 A.U. in group B, 210 ± 16 A.U. in group C, 192 ± 28 A.U. in group D, 214 ± 21 A.U. in group E and 161 ± 23 A.U. in group F. The LLC-Gen formulation proved to diminish the degree of erythema slightly, but when electroporation was applied the LLC-Gen formulation acted more effectively. On day 21 of the experiment, the levels were 59 ± 3 A.U. in group A, 228 ± 14 A.U. in group B, 215 ± 4 A.U. in group C, 183 ± 14 A.U. in group D, 228 ± 3 A.U. in group E and 152 ± 9 A.U. in group F. Significant results (*p ˂* 0.05) between the different experimental groups were found as shown in Figure 5.

**Figure 6:**
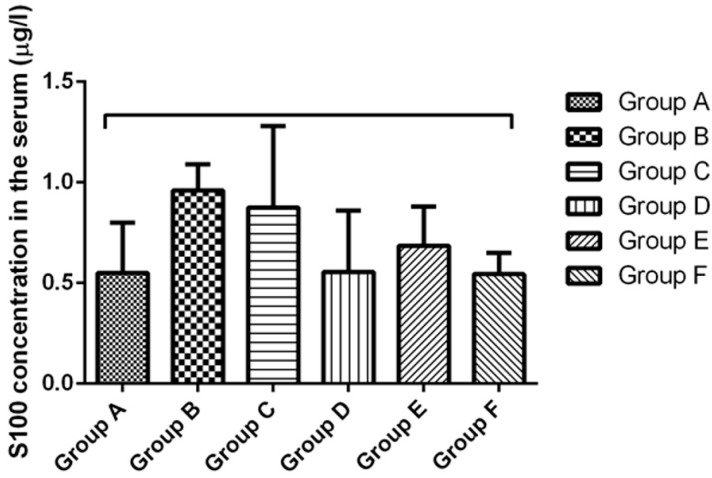
Serum concentrations of the protein S100B (µg/L) in the different experimental groups on day 21 of the experiment.

**Figure 7:**
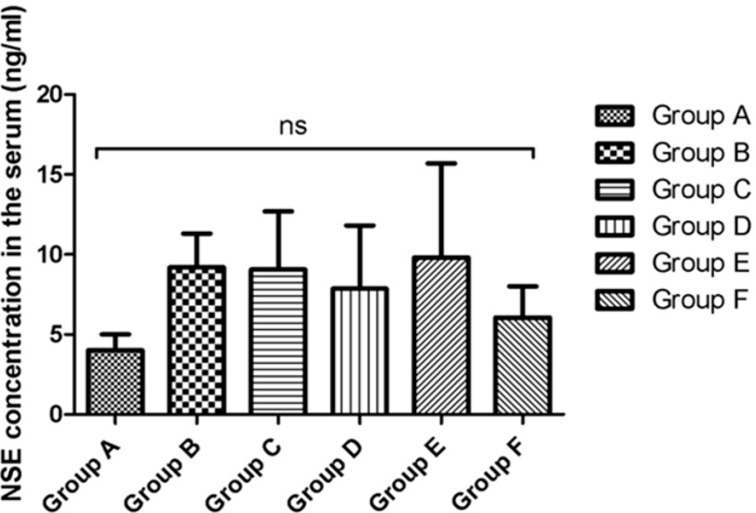
Serum neuron specific enolase (NSE) concentrations (ng/mL) in the different experimental groups on day 21 of the experiment. “ns” indicates not-significant.

On day 21 of the experiment, blood was collected from the mice in all groups and the serum concentrations of the protein S100B (µg/L) (a highly specific melanoma marker) and of neuron specific enolase (NSE) (ng/L) were determined. The mean concentrations of S100B in the different experimental groups were 0.55 ± 0.35 µg/L in group A, 0.96 ± 0.18 µg/L in group B, 0.86 ± 0.56 µg/L in group C, 0.510 ± 0.36 µg/L in group D, 0.690 ± 0.28 µg/L in group E and 0.54 ± 0.28 µg/L in group F. Higher values than the control were observed in the groups inoculated with melanoma cells. Although the mean values were slightly lower when electroporation was applied, the LLC-Gen formulation did not have a significant effect on the serum S100B concentration (Figure 6). The mean concentrations of NSE in the different experimental groups were 4.0 ± 1.4 ng/L in group A, 9.2 ± 3.0 ng/L in group B, 9.0 ± 5.1 ng/L in group C, 7.9 ± 5.6 ng/L in group D, 9.8 ± 8.3 ng/L in group E and 6.0 ± 2.8 ng/L in group F. Again, levels higher than the control were observed in the groups inoculated with melanoma cells. Although the mean NSE levels were slightly lower when electroporation was applied, the LLC-Gen formulation did not have a significant effect in reducing this marker either (Figure 7).

### 2.3. Histological and Immunohistochemical Results

**Figure 8:**
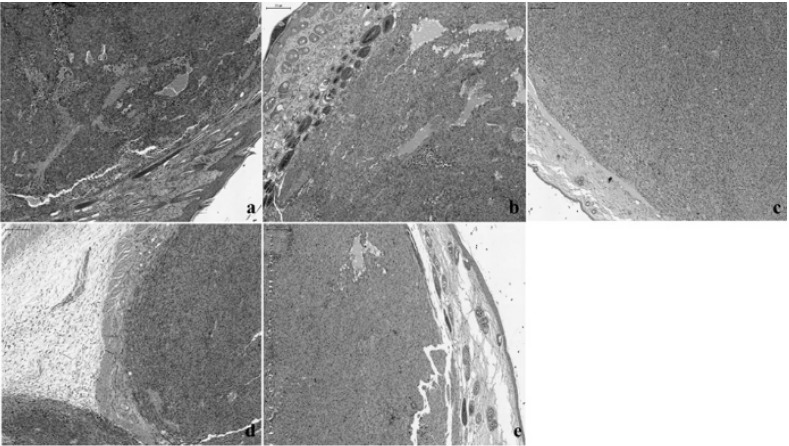
Hematoxylin–eosin (HE) staining in the different groups with skin melanoma: (**a**,**b**): groups B and C with moderate to intense pigmentation in almost all the tumor cells; (**c**): group D, exhibiting melanoma with weak and isolated pigment; (**d**): group E, displaying moderate pigmentation, with intense pigmentation in a few nests of tumor melanocytes; (**e**): group F, with the presence of melanin in isolated cells. Magnification: 40×; scale bar: 200 μm.

Conventional hematoxylin–eosin (HE) analysis revealed similar features in groups B and C, with moderate to intense pigmentation in almost all the tumor cells (Figure 8a,b). In group D, weak pigmentation was observed in isolated cells (Figure 8c). Group E was characterized by moderate pigmentation with intense pigmentation in a few nests of tumor melanocytes (Figure 8d). Group F exhibited similarities with group D, with a general feature of a lower level of pigmentation and with melanin present in a few tumor cells (Figure 8e).

**Figure 9:**
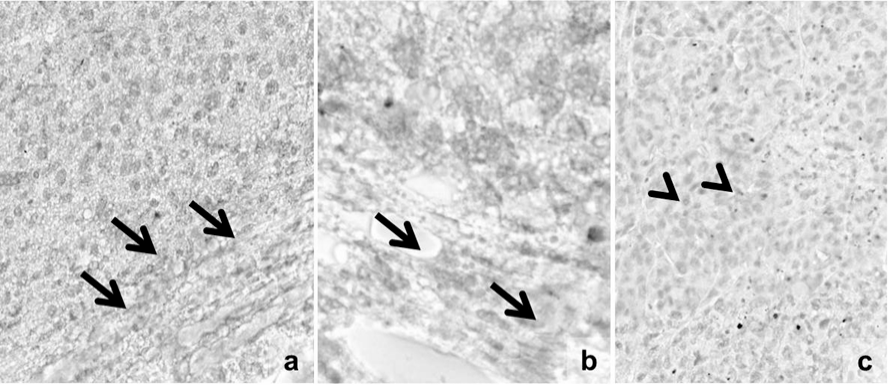
Representative pictures of platelet-derived growth factor receptor β (PDGFRβ)-immunostaining. Only a mild intratumoral reaction is seen in the peritumoral stromal myofibroblasts (**a**,**b**) and in the tumor cells (**c**), independently of the nature of the groups. Arrows and arrowheads highlight peritumoral stromal cells and intratumoral positive tumor cells, respectively. Dacarbazine red reaction; optical scanning microscopy—OM 200×.

In the case of platelet-derived growth factor receptor β (PDGFRβ) immunostaining only a mild intratumoral reaction was seen in the peritumoral stromal myofibroblasts (Figure 9a,b) and in the tumor cells (Figure 9c), independently of the nature of the groups.

### 2.4. Discussion

Polarization microscopy can be used for the examination and identification of the LLC system. The behavior of light in crystalline materials depends on its direction relative to the crystal structure. Unlike isotropic materials which are invisible between the crossed polarizers of a polarization microscope, a thin sample of LLC in the same experimental arrangement yields a typical picture (called texture) which shows the effective positional birefringence of the system [21].

Oscillating rheological measurements offer a convenient possibility for identification of the lamellar phase in dermal and transdermal drug delivery systems [22]. The viscoelastic properties are indicated by the changes in the elastic storage modulus (G′), and the viscous loss modulus (G″), as a function of frequency. The G′, which defines the energy stored per unit volume, is proportional to the elastic property of the sample, whereas the G″ is the energy dissipated per unit volume, proportional to the viscous component of the sample [23,24]. It is clear from Figure 2 that the blank LLC composition demonstrates more elastic than viscous behavior. The incorporation of Gen led to increases in G′ and G″, with maintenance of LLC state.

Gen has previously been described as a phytocompound active on melanoma, both on different cell lines *in vitro* and on various murine models *in vivo* [25,26,27,28]. An half maximal inhibitory concentration (IC_50_) of 12.5 µM has been reported after seven days of incubation in the case of the B164A5 cell line for the induction of angiogenesis and the inhibition of solid tumors when the cell line was implanted in C57BL6J mice, at a rate of 30%–50% (depending on the mode of administration) [13,29,30]. The present study involves a new approach toward the recovery of murine melanoma lesions: electroporation of an LLC-Gen formulation. This approach combines a new local formulation and a new mode of administration for Gen. To the best of our knowledge, the effect of the local administration of a Gen-based formulation on murine melanoma has not been reported previously. We chose this combination because LLCs have been intensively studied as carrier systems for lipophilic drugs with a view to increasing the dermal penetration [14,31,32], and because electroporation increases the cutaneous penetration of various active agents [33,34,35]. We used polarizing microscopy and rheological investigations to confirm that this system was a lamellar LLC.

Our first important observation was that the tumors appeared two days later in the mice treated with LLC with 3% Gen and electroporated for six minutes at high-voltage than in the untreated or the normally treated mice. The second important observation was that the locally applied formulation decreased the tumor volume to an extent proportional to the number of days of treatment, and when electroporation was applied, the effect was increased.

Experimental data suggest that the degree of skin pigmentation correlates directly with the amount of melanin [36]. However, the measurement of skin pigmentation is not a perfectly reliable method to determine the exact melanin content but it indicates the relative melanin pigmentation, as a prediction value [4,37]. The decrease in skin pigmentation can reflect with certain limitation the decrease of melanin content reflecting the inhibition of melanogenesis, as a secondary effect. Substantial evidence indicates that the inhibition of melanogenesis has a therapeutic effect [38,39,40,41]. The presence of melanin pigment or the presence of an active melanogenesis can alter the sensitivity and behavior of melanoma cells to chemoimmunotherapy [38,39], thus the inhibition of melanogenesis can represent a therapeutic target. Melanin measurement with the Mexameter MX18 in physiological or pathological cases, with or without treatment is a noninvasive method currently used in skin research [42,43,44,45]. The level of melanin in the mice treated with LLCs with 3% Gen and electroporated for 6 min at high-voltage was significantly decreased. The same held for the extent of erythema, a skin disorder that occurs with any skin lesion [13]. Melanoma is one of the most severe skin lesions [46].

After the noninvasive measurements, blood was collected in order to determine the concentrations in the different experimental groups of two melanoma markers: S100B and NSE. S100B is a 21-kDa protein that plays a role in preserving the cytoskeletal integrity, migration, cell cycle regulation and apoptosis [47]. Elevated S100B serum levels have been found in advanced stages of melanoma and have been associated with tumor progression and a shorter survival [48]. In contrast with S100B, NSE has been reported to be elevated in the early stages of melanoma [49]. Our results revealed decreased serum concentrations of both selected markers in mice treated with LLCs with 3% Gen and electroporated for 6 min at high-voltage as compared with the untreated mice, but the results were not statistically significant (*p ˃* 0.05).

The PDGFRs are members of a family of tyrosine kinase receptors found on the surface of cells. They regulate important processes such as cell differentiation, proliferation, growth and development [50]. There are two forms, encoded by different genes, PDGFRα and PDGFRβ [51]. PDGFRβ is normally present in cells of mesenchymal origin, e.g., vascular endothelial cells, fibroblasts, bone marrow cells and monocytes [52]. However, its expression is upregulated in the event of imbalances such as atherosclerosis, fibrosis and cancer [50]. B16 murine melanoma cells express PDGFRβ in the tumor microenvironment and the tumor stroma [53,54]. We therefore checked its expression after local Gen treatment with or without electroporation. The results showed that there was no marked difference in expression profile between the groups. Recent studies have shown that, in the case of the vascular smooth muscle, isoflavone inhibits PDGF-stimulated proteoglycan synthesis without blocking PDGFRβ phosphorylation [55]. Additionally, Gen did not affect early signal transduction through PDGFRβ in primary cultured rat aortic smooth muscle cells [56]. We can now report that Gen did not affect the expression of PDGFR and PDGFRβ in B16 murine melanoma.

## 3. Experimental Section

### 3.1. Materials

Gen was from Extrasynthèse (Genay, France; purity > 95%), and the mouse adherent melanoma cell line B164A5 was purchased from the European Collection of Cell Cultures (ECACC) (Salisbury, UK). The cells were grown in Dulbecco’s Modified Eagle’s Medium (DMEM) supplemented with 10% heat-inactivated fetal calf serum (FCS), 1% non-essential amino acids and 1% penicillin-streptomycin in a humidified atmosphere containing 5% CO_2_ at 37 °C. All cell culture media and supplements were from Life Technologies (Paisley, UK). In the preparation for injection, cells were trypsinized, counted with the aid of trypan blue, washed with PBS, resuspended at 1 × 10^6^ cells/0.1 mL in saline solution and injected immediately, as described below.

### 3.2. Lamellar Lyotropic Liquid Crystal Genistein (LLC-Gen) Formulation

The lamellar LLC formulation was developed because this crystal structure demonstrates the greatest similarity to the lipid bilayer of the skin [57]. Three percent of Gen was incorporated as active agent. The carrier was a mixture of a non-ionic surfactant, Cremophor RH40 (Polyoxyl 40 Hydrogenated Castor Oil USP/NF), obtained from BASF (Ludwigshafen, Germany). It is tolerated well by tissues and has low toxicity, and its HLB (hydrophilic-lipophilic balance) value is 14–16. The aqueous phase of the systems was purified water (European Pharmacopoeia 6th Edition), while the oil phase was isopropyl myristate (Merck, Budapest, Hungary).

A Gen-free LLC was prepared by the following procedure. The oil-surfactant mixture (oil:surfactant ratio = 2:1) was homogenized with a magnetic stirrer at room temperature. Ten percent of water was then added in small amounts to this mixture during stirring. A similar composition was prepared by using Gen, and the 3% of Gen was incorporated in the oil-surfactant mixture with a magnetic stirrer.

### 3.3. Electroporation Parameters

The Mezoforte Duo Mez 120905-D was provided by the Derm Clinic of Anti-Aging Dermatology, Aesthetic Laser and Plastic Surgery (Budapest, Hungary). The device operates on the basis of a pulsed electromagnetic field. The polypropylene-covered treating handpiece contains a 25 mm diameter plate electrode. Modulation was achieved with 1800 V high-voltage pulses with a voltage pulse duration of 5 ms followed by a 20 ms break. The treatment time was 6 min.

### 3.4. Polarization Microscopic Examinations

The structures of the samples were examined with a polarization microscope (LEICA Q500 MC Image Analyzer System, Leica Microsystems GmbH, Wetzlar, Germany) at room temperature. The magnification was 20×.

### 3.5. Rheological Investigations

The rheological profiles of the samples were studied with a PaarPhysica MCR101 rheometer (Anton Paar GmbH, Graz, Austria). The measuring device was of plate-plate type (diameter 25 mm, gap distance 0.2 mm). The measurements were carried out at 25 °C. The linear viscoelastic range was determined in the first step by examining the complex modulus as a function of the shear stress at a given frequency (1 Hz). On the basis of these experiments, the shear stress was set at 1 Pa during the dynamic testing as this value was always within the linear viscoelastic range. The storage and loss moduli values were examined as a function of frequency (0.01–10 Hz).

### 3.6. Animal Studies

Animal studies were conducted on 7–8 week old C57BL/6J female mice with an average weight of 20–25 g, purchased from Charles River (Sulzfeld, Germany). The working protocol strictly followed all the National Institute of Animal Health (NIAH) regulations: animals were maintained under standard conditions throughout the experiments: a 12 h light-dark cycle, food and water *ad libitum*, temperature 24 °C and humidity >55%. The experiments were conducted in accordance with the rules of the Ethical Committee of University of Medicine and Pharmacy “Victor Babes” Timisoara, Romania no. 27.11.2014_ref.1.2015. The number of mice involved in the study was 30, divided equally into 6 groups of 5 animals as follows:

Group A: blank group;

Group B: mice inoculated with B164A5 cells and otherwise not treated;

Group C: mice inoculated with B164A5 cells and treated with LLCs without Gen;

Group D: mice inoculated with B164A5 cells and treated with LLCs with 3% Gen;

Group E: mice inoculated with B164A5 cells and treated with LLCs without Gen and electroporated at high-voltage for 6 min with a Mezoforte Duo Mez 120905-D;

Group F: mice inoculated with B164A5 cells and treated with LLCs with 3% Gen and electroporated at high-voltage for 6 min with a Mezoforte Duo Mez 120905-D.

On day 0 of the experiment, the mice in groups B–F received a subcutaneous (s.c.) inoculation of 0.1 mL containing 1 × 10^6^ cells/mouse into the depilated lateral abdomen. For the mice in groups C–F treatment with 2 mL of a 3% Gen-LLC formulation was administered from day 2 post-inoculation, using 6 min of high-voltage electroporation (Mezoforte Duo Mez 120905-D) or the classical application as described above. The mice were inspected daily for the development of tumors or other changes. Tumor growth was measured daily in millimeters, using calipers, and the tumor volume was estimated by the formula: length × width^2^/2. On day 21 post-inoculation, after the last measurements had been made, the mice were anesthetized with gaseous isoflurane. Blood was collected from the cava vein, and the concentrations of the melanoma-specific markers S100B and NSE (neuron specific enolase) were investigated. The mice were subsequently euthanized by cervical dislocation. Tumors were collected, measured and weighed, and histological and immunohistochemical analyses were performed.

### 3.7. Noninvasive Skin Measurements

All the measurements on the skin of the mice were carried out with a Multiprobe Adapter System (MPA5) from Courage-Khazaka, Köln, Germany: the measurements of pigmentation and erythema with the Mexameter^®^ MX 18 from Courage-Khazaka, Köln, Germany furnish quantitative information relating to the relative melanin pigmentation and erythema (hemoglobin) subject to modifications by tumor evolution with or without treatment.

The measurements were made on four regions of the skin located near the tumor, and the mean and standard deviation were calculated. The amounts of melanin and erythema were measured at the baseline (day 0) and then every two days until day 21 of the experiment. The measurement areas were 5 mm in diameter.

### 3.8. Determination of Neuron Specific Enolase (NSE) and S100 Calcium Binding Protein B (S100B) in the Blood

The mice were anesthetized with isoflurane, and blood was collected from the posterior vena cava and by cardiac puncture and inserted into a serum separator tub. Samples were allowed to clot overnight at 4 °C before centrifugation at approximately 1000× *g* for 20 min. The enzyme-linked immunosorbent assay kits for S100 calcium binding protein B (S100B) and neuron specific enolase (NSE) were from Cloud Clone Corp. (Houston, TX, USA). The manufacturer’s protocol was used to determine the S100B and NSE concentrations in the mouse serum. All kit components and samples were brought to room temperature (18–25 °C) before use.

### 3.9. Histology

For histological analysis, tissue samples (skin) were fixed in 4% buffered formaldehyde solution and embedded into paraffin. For standardized evaluation, each sample was organized into a tissue microarray (TMA) block. Sections of 4 um from the TMA blocks were deparaffinized, stained with hematoxylin-eosin (HE) by a Leica Autostainer, Medist, Bucharest, Romania and analyzed microscopically. Samples were visualized and archived by computerized scanning microscopy (3D Histech Ltd., Budapest, Hungary).

### 3.10. Immunohistochemistry

Sections of 4 µm from the TMA blocks were used for immunohistochemistry. Rabbit antibody of PDGFRβ (polyclonal; Sigma-Aldrich Co., St. Louis, MI, USA) was applied in a dilution of 1:2000. Antigen retrieval was performed according to the datasheets of the antibody. Visualization was carried out with an autostainer with the Leica Bond kit. Sections were counterstained with conventional hematoxylin and gently coverslipped. Evaluation was based on the brown colorimetric discoloration of the samples. For intensity assessment, optical densitometry was performed with Image Pro Plus software, Media Cybernetics, Rockville, MD, USA.

### 3.11. Statistics

Results are presented as means ± SD. One-way ANOVA followed by the Bonferroni test was used to determine the statistical differences between the various experimental and control groups; *, ** and *** indicate *p* < 0.05, *p* < 0.01 and *p* < 0.001. “ns” indicates not-significant.

## 4. Conclusions

We conclude that LLCs serve as a good formulation for the local delivery of genistein. Further studies will be conducted with higher concentrations of this active agent. Electroporation proved to be a good method for the delivery of the new formulation because it delayed the appearance of the tumors, and decreased the tumor volume, the amount of melanin and the degree of erythema. HE staining confirmed the above events, but this approach did not have a significant effect in terms of the serum concentrations of S100B and NSE, or the tissue expression of the PDGFRβ antibody.
